# The Prevalence of Exclusive Breastfeeding Practice and Its Predictors in the First Six Months of Life Among Working Mothers in Riyadh, Saudi Arabia

**DOI:** 10.7759/cureus.58729

**Published:** 2024-04-22

**Authors:** Fatimah Y Aljawoan, Alanoud I Alabdulkarim, Almaha A Alhumaidan, Renad Alsaeed, Layan M Aldosari

**Affiliations:** 1 Obstetrics and Gynecology, Dent Medical Center, Riyadh, SAU; 2 Medicine, Imam Mohammad Ibn Saud Islamic University, Riyadh, SAU; 3 General Surgery, Imam Mohammad Ibn Saud Islamic University, Riyadh, SAU; 4 Medicine and Surgery, Imam Mohammad Ibn Saud Islamic University, Riyadh, SAU; 5 Gastroenterology, Imam Mohammad Ibn Saud Islamic University, Riyadh, SAU

**Keywords:** workplace, working mothers, prevalence, breastfeeding, riyadh, exclusive breastfeeding

## Abstract

Introduction

The United Nations Children's Fund (UNICEF) and the World Health Organization (WHO) recommendations are early initiation of breastfeeding and exclusive breastfeeding (EBF) for the first six months of infants' lives. Despite the WHO and UNICEF recommendations and expanding evidence of the significance of exclusive breastfeeding, about two-thirds of infants worldwide have not received exclusive breastfeeding for the six recommended months. This study aims to estimate the prevalence of working mothers exclusively breastfeeding in the first six months of infants’ lives and investigate their predictors in Riyadh, Saudi Arabia.

Methods

A cross-sectional community-based study was conducted for four months in 2022. The study included working mothers who have a child in the age range of 6-24 months living in Riyadh. Data was collected through an online questionnaire and analyzed using the Statistical Package for the Social Sciences (SPSS) version 29 (IBM SPSS Statistics, Armonk, NY) program.

Results

A sample of 118 participants were included in the study. Their prevalence for EBF practice for the recommended period is 28% (n=33). Around 58.5% (n=69) of the participants did not receive breastfeeding counseling during antenatal visits. Almost half the infants were given prelacteal feeding. Male infants are two times more likely to be exclusively breastfed for the recommended period than female infants. Work-related pressures were a key factor in the discontinuation of breastfeeding (53.4%, n=63).

Conclusion

This study highlights the lack of breastfeeding counseling and breastfeeding work regulation, alongside concerns about colostrum avoidance and prelacteal feeding. While EBF rates show progress, delayed initiation and work-related pressures remain challenges. Gender disparity in exclusive breastfeeding urges targeted interventions for more equitable outcomes.

## Introduction

Breastfeeding, according to the World Health Organization (WHO) and the United Nations Children's Fund (UNICEF), is the practice of feeding a baby or infant with breast milk directly from the mother's breast [1]. It is recommended as the optimal feeding method for infants up to six months of age, with continued breastfeeding alongside appropriate complementary foods up to two years of age or beyond [1]. The recommendations from the United Nations Children's Fund (UNICEF) and World Health Organization (WHO) emphasize the importance of early initiation of breastfeeding, urging mothers to place their infants to the breast within the first hour of life [1]. Additionally, they advocate for exclusive breastfeeding (EBF) during the initial six months, meaning infants should receive only breast milk, excluding water [1]. After this period, complementary foods can be introduced alongside breast milk for up to two years and beyond [1]. Breast milk is described as the ideal food for infants, meeting their needs comprehensively during the first six months, half of their needs in the subsequent six months, and about a third during the second year [1].

Colostrum, the initial breast milk produced in the early days of an infant's life, is highlighted for its richness in antibodies and nutrients, providing crucial protection for newborns [1,2]. Breastfeeding has been shown to contribute to higher intelligence in children, reduce the risk of obesity and diabetes, and offer health benefits to mothers, including a decreased risk of breast and ovarian cancers [2].

In Riyadh, Saudi Arabia, as of the end of 2019, the number of employed women was 585,838, constituting 17.7% of total employees in Riyadh [3]. Most jobs in Saudi Arabia allow for a paid 10-week maternity leave, starting four weeks before the due date, followed by an unpaid month off [4]. This structure often necessitates mothers to return to work before their infants reach the recommended six months of exclusive breastfeeding [4]. Lactating women face challenges in maintaining breastfeeding practices due to a lack of suitable places or time to express milk, leading to the risk of premature weaning [5]. Alzaheb et al. (2017) identified high-risk groups including working mothers and those unaware of current breastfeeding recommendations [6]. The duration of breastfeeding for working mothers is linked to their ability to pump or nurse at the workplace, emphasizing the importance of regulations to support breastfeeding during work hours [5].

Globally, about 21% of children in developed countries have never been breastfed, while this figure is only 4% in low- and middle-income countries [1]. The Eastern Mediterranean Regional Office of the World Health Organization (WHO) reports high rates of early breastfeeding initiation, exceeding 60% [7]. Cesarean delivery and low birth weight are associated with lower rates of exclusive breastfeeding at six months compared to normal birth conditions [6]. Improper advertising of alternatives to human milk has negatively impacted breastfeeding rates worldwide [1]. Promoting and supporting breastfeeding are crucial for saving the lives of infants and children, making it the most significant preventative action [8].

The current study aims to assess the prevalence of exclusive breastfeeding among working mothers in Riyadh, Saudi Arabia, during the first six months of their infants' lives. The objectives include estimating the prevalence and mean duration of exclusive breastfeeding, identifying barriers to initiation or continuation, and investigating predictors influencing the initiation and duration of exclusive breastfeeding among working mothers.

## Materials and methods

Study design and sample

This cross-sectional community-based study was conducted in Riyadh between February and June 2022. Riyadh is the capital city of Saudi Arabia, with a population of 8,591,748 [3]. The inclusion criteria were working mothers who have a child in the age range of 6-24 months living in Riyadh. The exclusion criteria aim to identify factors that could potentially confound the promotion or inhibition of exclusive breastfeeding. These criteria may include medical conditions in the mother or infant that could affect breastfeeding ability or safety, such as certain infections or metabolic disorders. Additionally, socioeconomic factors, such as lack of access to supportive resources or cultural practices that discourage breastfeeding, may also be considered as exclusion criteria. Our exclusion criteria included any child younger than six months, a child older than 24 months, a participant living outside of Riyadh, a participant being unemployed, or male. Any response that did not meet the previously mentioned criteria was excluded, for example, male participants, women living outside of Riyadh, and women with children who do not meet the age criteria (6-24 months). The sample size was calculated to be 107 with a confidence level of 90% using an 11% prevalence. We calculated the prevalence by the following data: the total number of females in Riyadh is 3,209,408 [3] and the number of females who give birth in Riyadh is about 90,000 yearly [3], which is 2.8% out of the total number of females in Riyadh. Furthermore, the percentage of working females to the total number of females in Riyadh is about 20%. So, we estimated the prevalence of working females in Riyadh with a child aged 6-24 months to be about 11%. The sampling technique used was convenience sampling.

Ethical approval

The study received ethical approval from the Research Ethics Committee at Imam Mohammad Ibn Saud Islamic University, Riyadh, Saudi Arabia (reference number: 96/2021).

Data collection method and analysis

Data was collected through a self-administered Google Forms (Google, Inc., Mountain View, CA) questionnaire distributed to the mothers through a few daycares in Riyadh and social media applications WhatsApp and Twitter. The questionnaire focuses on providing basic demographic information in addition to feeding patterns, initiation, and duration of exclusive breastfeeding, alongside questions concerning the beliefs and barriers facing participants, and it consists of five sections. The first section assesses the basic sociodemographic characteristics. Moreover, this section contains excluding questions to ensure the data collected was from the selected population. The second section assesses information about the child under evaluation, which includes 13 questions in addition to the infant's age, which was used for sample exclusion. The third section assesses obstetric history and health service-related factors. The fourth section assesses information regarding the breastfeeding practice of the current child. This section includes nine questions regarding the time and place of breastfeeding initiation, the practice of exclusive breastfeeding (EBF), duration of exclusive breastfeeding, family support, baby caretaker, received colostrum, prelacteal feeding, and the mother's satisfaction with the duration she breastfed. The fifth section assesses awareness and attitudes about breastfeeding.

The study investigated the predictors of maintaining exclusive breastfeeding for six months for working mothers. The independent variables in this study were sociodemographic characteristics, child variables, work environment, obstetric history, breastfeeding practice, childcare, support, awareness, and attitudes. The dependent variable was completing six months of exclusive breastfeeding. Exclusive breastfeeding for six months was defined as receiving only breast milk without other food or water in the initial six months of life. Analysis was done using the Statistical Package for the Social Sciences (SPSS) version 29 (IBM SPSS Statistics, Armonk, NY). The data was expressed as percentages. The relationship between categorical variables was analyzed using Chi-square tests. P-value < 0.05 was considered significant.

## Results

The questionnaire received a total of 284 responses. A total of 118 remained after excluding 166 responses that did not apply to the study inclusion criteria. One hundred one responses were excluded due to the age of the child being less than six months or more than 24 months. Thirty-eight non-working women submitted a response and therefore were excluded. Furthermore, 25 males and two non-Riyadh residents were also excluded.

Characteristics of the study sample

Table 1 shows the characteristics of the participants in the study. The majority were married (92.4%, n=109), were Saudi nationals (98.3%, n=116), and lived in the north of Riyadh (44.9%, n=53). The mean age of mothers was 34 years with a standard deviation of seven years. Both mothers and fathers' education were mostly university or higher (87.3%, n=103, and 81.4%, n=96, respectively). The mean family income was 12,258.5 SR/month with a standard deviation of 3,760.9 SR. About half of the sample worked in education (47%, n=56). Regarding the gender of the child, 41.5% (n=49) were females and 58.5% (n=69) were males. The mean age of the children was 11.5 years with a standard deviation of 4.7 years. The majority had normal birth weight (88.1%, n=104). Most had an employed father (95.8%, n=113). For 35.6% (n=42) of the sample, this was their first child. About a third had two or more children under five years old (33.9%, n=40), and 61.9% (n=73) had one or less children above five years old.

**Table 1:** Characteristics of the study sample (N=118)

Variable	Number	%
Age (years)		
20-29	35	29.7
30-39	60	50.8
>40	23	19.4
Place of residence		
East of Riyadh	35	29.7
North of Riyadh	53	44.9
South of Riyadh	16	13.6
West of Riyadh	14	11.9
Marital status		
Divorced	6	5.1
Married	109	92.4
Widowed	3	2.5
Nationality		
Non-Saudi	2	1.7
Saudi	116	98.3
Mother's education		
High school/below	15	12.6
University/higher	103	87.3
Father's education		
High school/below	22	18.6
University/higher	96	81.4
Father's employment		
Employed	113	95.8
Student	1	0.8
Unemployed	4	3.4
Family income (Saudi Riyal)		
<3,000	6	5.1
3,000-4,999	4	3.4
5,000-9,999	18	15.3
10,000-14,999	29	24.6
≥15,000	61	51.7
Working profession		
Military, security	2	1.6
Business and banking	7	5.9
Management	19	16.1
Law	3	2.5
IT and engineering	9	7.6
Education	56	47.5
Healthcare	19	16.1
Student	3	2.5
Sex of the child		
Female	49	41.5
Male	69	58.5
Birth order of the child		
First born	42	35.6
Second born	31	26.3
Third and above	45	38.1
Age of the child (months)		
6	14	11.9
7	18	15.3
8	11	9.3
9	12	10.2
10	12	10.2
11	3	2.5
12	10	8.5
12-24	38	32.2
Number of children above 5 years		
≤1	73	61.9
≥2	45	38.1
Number of children under 5 years		
≤1	78	66.1
≥2	40	33.9
Child's birth weight		
Normal	104	88.1
Overweight	3	2.5
Underweight	11	9.3
Work environment		
Gender mixed	43	36.4
Not gender mixed	75	63.6
Working hours/day		
>10 hours	2	1.7
10 hours	5	4.2
6 hours	56	47.5
8 hours	55	46.6
Night shifts		
Yes	16	13.6
No	102	86.4
Work from home		
No	102	86.4
Yes	16	13.6

More than half of the women worked in a female-only work environment (63.6%, n=75). The mean working hours was 7.2 with a standard deviation of 1.2. Having night shifts was the least common (13.6%, n=102). A small number of women worked from home (13.6%, n=16).

Table 2 shows exclusive breastfeeding knowledge among working women in Riyadh. The majority has heard about exclusive breastfeeding and the expression of breast milk (66.9%, n=79, and 92.4%, n=109, respectively). Of the women, 68.6% (n=81) were aware of the definition of exclusive breastfeeding (EBF), and 43.2% (n=51) were aware of the recommended period. Most participants know that colostrum should be given (81.4%, n=96).

**Table 2:** Exclusive breastfeeding knowledge (N=118)

Variable	Number	%
Heard about exclusive breastfeeding		
Don't know	11	9.3
No	28	23.7
Yes	79	66.9
Heard about the expression of breast milk		
Don't know	2	1.7
No	7	5.9
Yes	109	92.4
Meaning of exclusive breastfeeding		
Breast milk and water only	22	18.6
Breast milk only	81	68.6
Breast milk with other supplementation	15	12.7
Recommended duration of exclusive breastfeeding		
1 month	15	12.7
12 months	13	11
24 months	39	33.1
6 months	51	43.2
What should be done with the colostrum		
Should be discarded	22	18.6
Should be given	96	81.4

Characteristics of natal care and breastfeeding

Table 3 shows the characteristics of natal care and breastfeeding. Private hospitals were the most common to receive antenatal care and delivery (49.2%, n=58, and 66.9%, n=79, respectively). About a tenth did not receive any antenatal care (9.3%, n=11). Furthermore, 58.5% (n=69) did not receive breastfeeding counseling during antenatal care visits. The majority had full-term (92.4%, n=109), followed by normal vaginal delivery (79.7%, n=94), and the majority did not require admission to the neonatal care unit (75.4%, n=89). About half received breastfeeding counseling immediately after delivery (57.6%, n=68). Only 30.5% (n=36) had a history of breastfeeding the previous child.

**Table 3:** Characteristics of natal care and breastfeeding (N=118) CS: cesarean section, BF: breastfeeding

Variable	Number	%
Facility where antenatal care was received		
Governmental	49	41.5
Private	58	49.2
No antenatal care	11	9.3
Breastfeeding counseling received during antenatal care visits		
Yes	49	41.5
No	69	58.5
Place of delivery		
Governmental	37	31.4
Private	79	66.9
Home	2	1.7
Mode of delivery		
CS	24	20.3
Vaginal delivery	94	79.7
Gestation		
Full term	109	92.4
Preterm	9	7.6
Admission to neonatal care unit		
Yes	29	24.6
No	89	75.4
Breastfeeding counseling received immediately after delivery		
Yes	68	57.6
No	50	42.4
History of breastfeeding the previous child		
Yes	36	30.5
No	82	69.5
Where was breastfeeding initiated?		
At home	22	18.6
At the hospital	96	81.4
Received colostrum?		
No	29	24.6
Yes	89	75.4
Prelacteal feeding?		
No	66	55.9
Yes	52	44.1
For how many months did you feed your children breast milk only?		
First 48 hours	13	11
1 month	21	17.8
3 months	16	13.6
6 months	17	14.4
More than 6 months	16	13.6
Never breastfeed	3	2.5
Practiced mixed feeding	32	27.1
Has the infant received water or formula milk during that period?		
No	39	33.1
Sometimes	29	24.6
Yes	50	42.4
Baby caretaker while mother is at work		
Daycare	15	12.7
Family	46	39
My child is with me at work	1	0.8
Mother	5	4.1
Nanny	42	35.6
Spouse	9	7.6
Family support of BF		
Most/all of the time	64	54.2
No support	19	16.1
Sometimes	35	29.7
Were you satisfied with the duration that you breastfed?		
No	23	19.5
Somewhat	33	28
Yes	62	52.5
Did you intend to breastfeed before you gave birth?		
No	12	10.2
Yes	106	89.8
Did you intend to breastfeed after returning from maternity leave?		
No	36	30.5
Yes	82	69.5
Do you think our medical practitioners' play a positive role in promoting exclusive breastfeeding?		
Don't know	21	17.8
No	46	39
Yes	51	43.2

The hospital was the most common place where breastfeeding was initiated (81.4%, n=96). One-quarter of the infants had not received colostrum (24.6%, n=29). Prelacteal feeding was given to 44.1% (n=52) of infants. Exclusive breastfeeding for six or more months had a prevalence of 28% (n=33). About a tenth were only breastfed for the first 48 hours(11%, n=13). Almost half fed the infant formula milk or water during the breastfeeding period (42.4%, n=50). The mean exclusive breastfeeding period was 2.3 months with a standard deviation of 2.5 months. Half had family support for breastfeeding (54.2%, n=64). The baby's caretaker while the mother was at work was mostly the family and the nanny (39%, n=46, and 35.6%, n=42, respectively). The percentage of women satisfied with their breastfeeding duration was 52.5% (n=62). Most had intended to breastfeed before giving birth (89.8%, n=106) and after returning from maternity leave (69.5%, n=82). Of the women, 43.2% (n=51) think that medical practitioners play a positive role in promoting exclusive breastfeeding.

Work and breastfeeding

As seen in Table 4, the mean maternity leave period was 8.8 weeks with a standard deviation of five weeks. Most returned to working full time postpartum (73.7%, n=87), and 28.8% (n=34) decreased their working hours after maternity. Half of the working mothers think that exclusive breastfeeding is not practically possible with work (50%, n=59). Tenth of the participants' workplaces encourage support of exclusive breastfeeding (EBF) practice (11.9%, n=14). Only 30.5% (n=36) have a daycare at work. Of the daycares, 33.3% (n=32) are not preserving and providing the mother’s milk properly, 37.5% (n=36) do not have a refrigerator to keep the milk, and 40.6% (n=39) do not have an appropriate place to breastfeed. Answers to having enough time to express milk at work were as follows: always, 5.1% (n=6); usually, 5.1% (n=6); sometimes, 11.9% (n=14); occasionally, 12.7% (n=15); never, 15.3% (n=18); and not applicable, 50% (n=59). Answers to having colleagues who are supportive of your milk expression are as follows: always, 8.5% (n=10); usually, 5.1% (n=6); sometimes, 13.6% (n=16); occasionally, 0.8% (n=1); never, 6.8% (n=1); and not applicable, 33.9% (n=40).

**Table 4:** Work and breastfeeding (N=118) EBF: exclusive breastfeeding

Variable	Number	%
Period of maternity leave		
Less than 4 weeks	16	13.6
4 weeks	18	15.3
6 weeks	14	11.9
10 weeks	46	39
3 months	17	14.4
6 months	7	5.9
Decrease working hours after maternity		
No	84	71.2
Yes	34	28.8
Do you think exclusive breastfeeding is practically possible with work?		
Don't know	18	15.3
No	59	50
Yes	41	34.7
Does your workplace encourage/support EBF practice?		
Don't know	24	20.3
No	80	67.8
Yes	14	11.9
Postpartum work schedule		
Did not return	6	5.1
Full time	87	73.7
Part time	25	21.2
Do you have a daycare at work?		
Don't know	3	2.5
No	79	66.9
Yes	36	30.5
If yes, then (1) does the daycare handle preserving and providing the mother's milk properly?		
Don't know	49	51
No	32	33.3
Yes	15	15.6
(2) Do they offer a refrigerator to keep your milk?		
Don't know	46	47.9
No	36	37.5
Yes	15	15.6
(3) Do they have an appropriate place for you to breastfeed your child?		
Don't know	42	43.8
No	39	40.6
Yes	15	15.6
Are you allowed to bring your baby to work?		
Don't know	13	11
No	87	73.7
Yes	18	15.3
Is there a suitable place for breastfeeding/pumping in your workplace?		
Don't know	10	8.5
No	92	78
Yes	16	13.6
Did you pump milk while working?		
No	90	76.3
Yes	28	23.7
Are you allowed nursing breaks?		
Don't know	20	16.9
No	71	60.2
Yes	27	22.9
Do you feel comfortable while breastfeeding at your workplace?		
No	54	45.8
Not applicable	51	43.2
Yes	13	11
Did you have enough time to express milk at work?		
Always	6	5.1
Never	18	15.3
Not applicable	59	50
Occasionally	15	12.7
Sometimes	14	11.9
Usually	6	5.1
Was the discontinuation of breastfeeding due to demands at work?		
No	55	46.6
Yes	63	53.4
Were your colleagues supportive of your milk expression efforts while working?		
Always	10	8.5
Colleagues did not know	37	31.4
Never	8	6.8
Not applicable	40	33.9
Occasionally	1	0.8
Sometimes	16	13.6
Usually	6	5.1

Of the mothers, 15% (n=18) are allowed to bring their babies to work. Most do not have a suitable place for breastfeeding or pumping in their workplace (78%, n=92). Furthermore, 23.7% (n=28) pump milk while working. Most are not allowed nursing breaks (60.2%, n=71). About a tenth are comfortable breastfeeding at their workplace (11%, n=13), and 43.2% (n=51) answered not applicable. Discontinuation of breastfeeding was mostly due to demands at work (53.4%, n=63).

Factors for practicing exclusive breastfeeding

**Figure 1:**
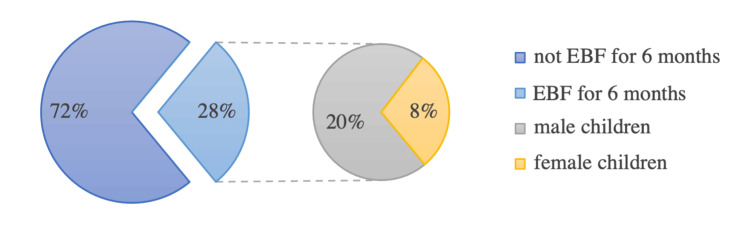
Prevalence of exclusive breastfeeding for the first six months, further divided by gender EBF: exclusive breastfeeding

The following tables elaborate on the categories with associations to exclusive breastfeeding. As seen in Table 5 and Figure 1, most exclusively breastfed children for the first six months were males (37.7%, n=26). The ratio of males to females exclusive breastfeeding (EBF) is 5:2 (14.3% females, n=7). Of the females, 22.5% (n=11) were only breastfed for the first 48 hours or never breastfed. On the other hand, the percentage for boys is 7.2% (n=5). Table 6 shows that mothers who practiced mixed feeding were the most unsatisfied with the period they breastfed (47.8%, n=11). The most satisfied were mothers who exclusively breastfed their child for six months or more (40.3%, n=14). Of the women who intended to breastfeed before giving birth, 29.3% (n=29.3) exclusively breastfed for six or more months (Table 7). As Table 8 shows, more than a third of mothers who intend to breastfeed after returning from maternity leave breastfed exclusively for six months or more (37.8%, n=31). Of the mothers who thought that exclusive breastfeeding is practically possible with work, 48.8% (n=20) exclusively breastfed for six months or more (Table 9). Table 10 shows that most mothers who worked the same hours after maternity exclusively breastfed for 1-3 months (31%, n=26), and most of those who had decreased working hours after maternity exclusively breastfed for six months or more (35.3%, n=12). Table 11 shows that 41% (n=26) of mothers who breastfed for 1-3 months discontinued breastfeeding due to demands at work.

**Table 5:** For how many months did you feed your children breast milk only? EBF: exclusive breastfeeding

EBF	Never	First 48 hours	1-3 months	6 months or more	Mixed feeding
Female (n=49)	4.1% (n=2)	18.4% (n=9)	36.7% (n=18)	14.3% (n=7)	26.5% (n=13)
Male (n=69)	1.4% (n=1)	5.8% (n=4)	27.5% (n=19)	37.7% (n=26)	27.5% (n=19)

**Table 6:** Were you satisfied with the duration that you breastfed? EBF: exclusive breastfeeding

EBF	Never	First 48 hours	1-3 months	6 months or more	Mixed feeding
No (n=23)	4.3% (n=1)	0% (n=0)	39.1% (n=9)	8.7% (n=2)	47.8% (n=11)
Somewhat (n=33)	3% (n=1)	6.1% (n=2)	30.3% (n=11)	18.2% (n=2)	39.4% (n=13)
Yes (n=62)	1.6% (n=1)	17.7 (n=11)	33.4% (n=17)	40.3% (n=14)	12.9% (n=8)

**Table 7:** Did you intend to breastfeed before you gave birth? EBF: exclusive breastfeeding

EBF	Never	First 48 hours	1-3 months	6 months or more	Mixed feeding
No (n=12)	16.7% (n=2)	0% (n=0)	41.7% (n=5)	16.7% (n=2)	25% (n=3)
Yes (n=106)	0.9% (n=1)	12.3% (n=13)	30.2% (n=32)	29.3% (n=31)	27.4% (n=29)

**Table 8:** Did you intend to breastfeed after returning from maternity leave? EBF: exclusive breastfeeding

EBF	Never	First 48 hours	1-3 months	6 months or more	Mixed feeding
No (n=36)	5.6% (n=2)	2.8% (n=1)	55.6% (n=20)	5.6% (n=2)	30.6% (n=11)
Yes (n=82)	1.2% (n=1)	14.6% (n=12)	20.8% (n=17)	37.8% (n=31)	25.6% (n=21)

**Table 9:** Do you think exclusive breastfeeding is practically possible with work? EBF: exclusive breastfeeding

EBF	Never	First 48 hours	1-3 months	6 months or more	Mixed feeding
No (n=59)	3.4% (n=2)	10.2% (n=6)	37.3% (n=24)	13.6% (n=8)	32.2% (n=59)
Don't know (n=18)	0% (n=0)	5.6% (n=1)	44.5% (n=8)	27.8% (n=5)	22.2% (n=4)
Yes (n=41)	2.4% (n=1)	14.6% (n=6)	12.2% (n=5)	48.8% (n=20)	22% (n=9)

**Table 10:** Decrease working hours after maternity EBF: exclusive breastfeeding

EBF	Never	First 48 hours	1-3 months	6 months or more	Mixed feeding
No (n=84)	2.4% (n=2)	11.9% (n=10)	31% (n=26)	25% (n=21)	29.8% (n=25)
Yes (n=34)	2.9% (n=1)	8.8% (n=3)	32.4% (n=11)	35.3% (n=12)	20.6% (n=7)

**Table 11:** Was the discontinuation of breastfeeding due to demands at work? EBF: exclusive breastfeeding

EBF	Never	First 48 hours	1-3 months	6 months or more	Mixed feeding
No (n=55)	1.8% (n=1)	10.9% (n=6)	20% (n=11)	40% (n=22)	27.3% (n=15)
Yes (n=63)	3.2% (n=2)	11.1% (n=7)	41% (n=26)	17.5% (n=11)	27% (n=17)

Table 12 shows that almost half of the women who had daycares at work that properly handled and preserved mothers' milk exclusively breastfed for six months or more (46.7%, n=7). Most women who had daycares at work that did not properly handle and preserve mothers' milk exclusively breastfed for 1-3 months (56.3%, n=18).

**Table 12:** Does the daycare at work handle preserving and providing the mother's milk properly? EBF: exclusive breastfeeding

EBF	Never	First 48 hours	1-3 months	6 months or more	Mixed feeding
No daycare at work (n=22)	0% (n=0)	18.2% (n=4)	23% (n=5)	18.1% (n=4)	40.9% (n=9)
Don't know (n=49)	4.1% (n=2)	8.2% (n=4)	20.4% (n=10)	32.6% (n=16)	34.7% (n=17)
No (n=32)	3.1% (n=1)	6.3% (n=2)	56.3% (n=18)	18.8% (n=6)	15.6% (n=5)
Yes (n=15)	0% (n=0)	20% (n=3)	16.6% (n=4)	46.7% (n=7)	6.7% (n=1)

Most women who had refrigerators at their daycare exclusively breastfed for six or more months (40%, n=6) (Table 13). Table 14 shows that 46.4% (n=13) of women who pumped while working exclusively breastfed for six or more months. Table 15 shows that 44.4% (n=12) of women who are allowed nursing breaks exclusively breastfed for six or more months. Table 16 shows that most women who work eight hours per day exclusively breastfed for 1-3 months (41.8%, n=23), while women who work six hours per day mostly practiced mixed feeding (32.1%, n=18). Women working from home had the same percentages of EBF for six or more months, 1-3 months, and the first 48 hours (31.3%, n=5) (Table 17). Table 18 shows that women working in a female-only workplace mostly practiced mixed feeding (32%, n=24), while those who worked in a gender-mixed environment mostly exclusively breastfed for six months or more (34.9%, n=15). 

**Table 13:** Does the daycare at work offer a refrigerator to keep your milk? EBF: exclusive breastfeeding

EBF	Never	First 48 hours	1-3 months	6 months or more	Mixed feeding
No daycare at work (n=21)	0% (n=0)	19% (n=4)	19% (n=4)	19% (n=4)	42.9% (n=9)
Don't know (n=46)	4.3% (n=2)	8.7% (n=4)	21.8% (n=10)	32.6% (n=15)	32.6% (n=15)
No (n=36)	2.8% (n=1)	2.8% (n=1)	55.6% (n=20)	22.2% (n=8)	16.7% (n=6)
Yes (n=15)	0% (n=0)	26.7% (n=4)	20% (n=3)	40% (n=6)	13.3% (n=2)

**Table 14:** Did you pump milk while working? EBF: exclusive breastfeeding

EBF	Never	First 48 hours	1-3 months	6 months or more	Mixed feeding
No (n=90)	3.3% (n=3)	6.7% (n=6)	34.4% (n=31)	22.2% (n=20)	33.3% (n=30)
Yes (n=28)	0% (n=0)	25% (n=7)	21.4% (n=6)	46.4% (n=13)	7.1% (n=2)

**Table 15:** Are you allowed nursing breaks? EBF: exclusive breastfeeding

EBF	Never	First 48 hours	1-3 months	6 months or more	Mixed feeding
Don't know (n=20)	0% (n=0)	10% (n=2)	20% (n=4)	15% (n=)	55% (n=11)
No (n=71)	4.3% (n=3)	8.5% (n=6)	38% (n=27)	25.4% (n=18)	23.9% (n=17)
Yes (n=27)	0% (n=0)	18.5% (n=5)	22.2% (n=6)	44.4% (n=12)	14.8% (n=4)

**Table 16:** Working hours/day EBF: exclusive breastfeeding

EBF	Never	First 48 hours	1-3 months	6 months or more	Mixed feeding
10 or more (n=7)	14.3% (n=1)	0% (n=0)	42.9% (n=3)	14.3% (n=1)	28.6% (n=2)
8 (n=55)	1.8% (n=1)	1.8% (n=1)	41.8% (n=23)	32.7% (n=18)	21.8% (n=12)
6 (n=56)	1.8% (n=1)	21.4% (n=12)	19.6% (n=11)	25% (n=14)	32.1% (n=18)

**Table 17:** Do you work from home? EBF: exclusive breastfeeding

EBF	Never	First 48 hours	1-3 months	6 months or more	Mixed feeding
No (n=102)	2.9% (n=3)	7.8% (n=8)	31.3% (n=32)	27.5% (n=28)	30.4% (n=31)
Yes (n=16)	0% (n=0)	31.3% (n=5)	31.3% (n=5)	31.3% (n=5)	6.3% (n=1)

**Table 18:** Is the work environment gender mixed? EBF: exclusive breastfeeding

EBF	Never	First 48 hours	1-3 months	6 months or more	Mixed feeding
No (n=75)	0% (n=0)	13.3% (n=10)	30.7% (n=23)	24% (n=18)	32% (n=24)
Yes (n=43)	7% (n=3)	7% (n=3)	32.6% (n=14)	34.9% (n=15)	18.6% (n=8)

## Discussion

The last two decades showed decreasing numbers of exclusive breastfeeding (EBF) despite the recommendations by the United Nations Children's Fund (UNICEF) and the World Health Organization (WHO) [1]. This study shows that the prevalence of working women practicing six months of EBF in Riyadh was 28% (n=33). Alyousefi (2021) reported the same result of 28% in Riyadh [9]. A lower prevalence was found in Taif, Saudi Arabia. Exclusive breastfeeding rate was 19% [10]. A higher percentage was shown among Mexican working mothers who continued to breastfeed for six months or more with 58% [11]. The current study highlights a big difference in the prevalence of EBF according to the gender of the infant. Males were predominantly exclusively breastfed for the recommended period in comparison to females. The prevalence for male infants was 37.7% (n=26). 

There is awareness of the definition of exclusive breastfeeding (EBF) (68.6%, n=81). However, knowledge of the recommended period was not in line with the recommendation of the World Health Organization (43.1%, n=51). Of the participants, 33.2% (n=39) chose 24 months for the EBF period, which could be due to the Islamic teaching of breastfeeding of up to two years of age. Half of the working mothers think that exclusive breastfeeding is impractical during work (n=59). Women who thought it was practically possible had higher rates of EBF. In the current study, almost two-thirds intended to continue breastfeeding after resuming work, and 37.8% (n=31) of them completed the recommended period of EBF. Women working in a female-only workplace mostly practiced mixed feeding (32%). Those working in a gender-mixed environment mostly practiced EBF for six months or more (34.9%, n=15).

Breastfeeding counseling during antenatal visits and after delivery has a positive influence over mothers. A study published in Hail revealed that most mothers received breastfeeding counseling during pregnancy and after delivery. Their exclusive breastfeeding prevalence was much higher than the current study (50.7%) [12]. Alshammari et al. [12] also mentioned a significant correlation between governmental antenatal care, history of breastfeeding the older child, receiving breastfeeding counseling after delivery, colostrum feeding to infants, and exclusive breastfeeding. In our study, only 41.5% (n=49) received breastfeeding counseling during antenatal visits, 57.6% (n=68) had breastfeeding counseling after delivery, and 30.5% (n=36) had a history of breastfeeding the older child.

The United Nations Children's Fund (UNICEF) and the World Health Organization (WHO) advise infants to start breastfeeding as soon as possible after delivery and to nurse exclusively for their first six months of life [1,2]. Our study shows that 81.4% (n=96) initiated breastfeeding in the hospital. A study showed that working mothers are 5.6 times less likely to initiate breastfeeding than housewives [10]. In Taif and Hail, 22% and 24% started instantly breastfeeding within one hour after birth [10,12]. A study published in 2021 analyzed the elements affecting initiation of breastfeeding in the group of working mothers; delay in breastfeeding was associated with mothers with older age, non-Arab nationality, cesarean section, non-rooming-in, and low birth weight [13].

Demanding work leaves around half of the mothers with no choice but to discontinue breastfeeding (n=63). In Southern Taiwan, breastfeeding was discontinued in 50.2% after returning to work, although 85% had accessible lactation rooms in their workplaces [14]. Some barriers to exclusive breastfeeding (EBF) continuation are failure to reach home or daycare to breastfeed through working hours, being unaware of how to express and store milk, and unavailability of lactation rooms and pumping tools [15].

According to Tsai [14], younger age, lower educational levels, clean room work, work shift, long hours, lack of awareness or use of breast pump break, and insufficient self-knowledge about breastfeeding are all undeniably connected with breastfeeding cessation after returning to work. Our study reveals that many struggled with not having a convenient place to breastfeed or pump in their workplace. Women who had nursing breaks at work and those who pumped milk in their workplace had higher rates to exclusively breastfeed for six months (44.4%, n=12, and 46.4%, n=13, respectively). A daycare was provided to one-third of the mothers at their workplaces (n=36). Almost half of the working mothers with daycares that handled their breast milk appropriately exclusively breastfed for the recommended period of the World Health Organization (n=7). This data suggests that implementing these amenities and regulations at the workplace aids in the continuation of exclusive breastfeeding for working women.

One limitation of our research is a small sample size. A larger sample size would be more representative of the population and enhance the generalizability of our findings. Depending solely on participants' self-reported answers introduces the possibility of memory and response distortions, which could impact the data's dependability. Hence, it is advisable to be cautious when extending the outcomes of the study to the broader populace.

## Conclusions

In summary, this study highlights breastfeeding practices and challenges of working mothers with six-month-old infants in Riyadh, Saudi Arabia. It reveals gaps in breastfeeding counseling during antenatal visits, the issue of prelacteal feeding, not receiving colostrum, and work-related pressures leading to breastfeeding discontinuation.

The study's finding of a 28%(n=33) prevalence of exclusive breastfeeding for six months in Riyadh provides valuable information for public health interventions and policies aimed at improving breastfeeding rates. The observed gender difference in exclusive breastfeeding, with male infants being predominantly breastfed for six months, underscores the need for tailored interventions to address potential disparities. Additionally, the mean exclusive breastfeeding period of only 2.3 months further emphasizes the need for comprehensive strategies. These strategies should address counseling, early initiation, workplace support, and gender-related nuances to promote optimal breastfeeding outcomes. Other studies with larger population sizes are needed. 
